# Intravenous fluid administration practice among nurses and midwives working in public hospitals of central Ethiopia: A cross-sectional study

**DOI:** 10.1016/j.heliyon.2023.e18720

**Published:** 2023-07-31

**Authors:** Million Teshome, Biftu Geda, Tesfaye Assebe Yadeta, Lema Mideksa, Meseret Robi Tura

**Affiliations:** a Department of Nursing, College of Medicine and Health Sciences, Ambo University, Ethiopia; b School of Public Health, College of Health Sciences and Medicine, Haramaya University, Ethiopia; c School of Nursing and Midwifery, College of Health Sciences and Medicine, Haramaya University, Ethiopia

**Keywords:** Nurse, Ethiopia, IVI, Intravenous fluid administration, Education

## Abstract

Intravenous fluid administration is the most common invasive procedure widely practiced in hospital settings. Globally, approximately 25 million people receive intravenous fluid therapy. Different factors affect nurse's intravenous fluid administration practices; that it may influences on the patient's outcome, increase morbidity and mortality. Previous study indicates that healthcare providers especially in developing countries have skills gap related to intravenous fluid administration. The purpose of this study was aimed to assess the intravenous fluid administration practices and its associated factors among nurses and midwives working in public hospitals of West Shewa zone, Central Ethiopia.

**Materials and methods:**

An institution-based cross-sectional study design was employed among 396 nurses and midwives in public hospitals in West Shewa zone, Central Ethiopia, from March 1 to 31, 2019. A Simple random sampling was used to select study participants using structured self-administered questionnaire, and observational checklist. The logistic regression model was used to identify association, and odds ratio was used to test the strength of the associations with outcome variable and predictor variables.

**Results:**

In this study, 59.3% (95%CI = 54.7%–64.5%) participants was had inadequate intravenous fluid administration practice. Inadequate knowledge (AOR 2.1; CI 95% = 1.36–3.36), being untrained (AOR 1.7; 95% CI = 1.04–2.86), unavailability of supervision (AOR 1.8; CI 95% = 1.14–2.99), and absence of incentives and promotion for nurses and midwives (AOR 2.1; CI 95% = 1.19–3.62) were significantly associated with outcome variable.

**Conclusion:**

Nearly seven in ten participants in the study setting were inadequate intravenous fluid practice. Inadequate knowledge, training, and absence of supervision by senior staffs, and absence of incentives and promotion for nurses and midwives were the main factors affecting intravenous fluid administration practice. Refresher courses, supervision, incentives and promotions were needed to nurses and midwives for an improvement of the intravenous fluid administration practice.

## Abbreviations

AGH: Ambo General Hospital
AICU: Adult Intensive Care Unit
AGH: Ambo General Hospital
AOR: Adjusted Odds Ratio
AURH: Ambo University Referral Hospital
BH: Bako Hospital
CI: Confidence Interval
COR: Crude Odds Ratio
CSA: Central Statistics Agency
FMOH: Federal Ministry of Health
GBH: Gindeberet Hospital
GeH: Gedo Hospital
GuH: Guder Hospital
IH: Inchin Hospital
JH: Jaldu Hospital
IHRERC: Institutional Health Research Ethical Review Committee
IVF: Intravenous Fluid; NICU, Neonatal Intensive Care Unit.

## 1 Introduction

Intravenous fluid therapy is the fastest way to deliver fluids or medications directly into a vein [1]. It is common aspect of therapy used for the dilution of medication and maintenance of body fluids, and widely used in hospital settings [2,3]. Maintenance of body fluid balance is a fundamental nursing practice as most of the patients admitted to hospitals require the administration of intravenous fluid as part of their medical management [4,5]. The type of fluids administered depends on their indication and composition. These may be required for fluid replacement, resuscitation, for the administration of medications or maintenance hydration. However, administration of wrong concentrations or types of intravenous fluid and errors in the rate of administration can cause clinical complications such as heart failure or volume depletion and can be fatal [5,6]. Almost these problems are common in every public hospital. Despite all these complications of intravenous fluid (IVF) imbalances, there are nurses and midwives still not practicing the correct way of fluid administration. So, it is important that nurses implement appropriate and correct IVF administration practices to provide safe patient care.

Intravenous fluid administration practices are important and remain an essential part of patients' care during hospitalization. Globally, approximately 25 million people receive intravenous (IV) therapy by the use of an intravenous cannula. It is a routine but potentially harmful procedure in hospitals [7,8]. Interventions studies carried out in the UK on intravenous fluid use in the acutely unwell adult medical inpatients have shown that the use of 0.9% sodium chloride solution compared to balanced fluid solutions is a factor for hospital mortality [5]. A study conducted on the completion of fluid balance charts on different wards of University Hospital in Australia indicated a gap in the complete documentation of the patients' fluid balance charts [9]. Another study was conducted in Pakistan on nurses' practice regarding IV fluid administration using observation checklist and the results showed that 35.10% do not meet the criteria to check the amount, type of fluid against doctor's orders and 36.06% do not meet criteria to accurately adjust the flow rate [10]. A similar study conducted at the University of Sree Chitra, Trivandrum found that only in 43.33% of the occasions the flow rate is accurately adjusted and in 50% situations the time in which the fluid started is documented [11]. Although IVF administration is the most common practice, no focus is given to its therapy as a medication. Hospitalized patients administered IVF for prolonged periods of time. Many IVF prescriptions are hastily written up. Most of the time nurses do not check the amount and type of fluid administered and at the same time they do not accurately adjust the flow rate. The major reasons for the poor practice were staff shortages, lack of training, lack of time and a perceived lack of importance for the practice. This can lead to inappropriate IVF composition, duration, documentation and, ultimately increased morbidity for patients and increase healthcare costs [9,[12], [13], [14]]. Adherences to the medication administration practice guidelines among nurses and midwives are also questionable and not addressed well. However, the Federal Ministry of Health (FMOH) of Ethiopia undertook initiatives to protect patients by setting standards and guidelines [11,15]. Different factors like adequate knowledge on IVF administration practices, availability of training, adequate staffs and times as well as supervision, work incentives, policy and guidelines are extremely playing a great role to achieve appropriate IVF administration practices [8,11,16].

Hence, it is important that nurses and midwives should know and use the recommended guidelines and protocols accordingly [17]. But, without adequately giving the due attention and assessing the current fluid administration practices of nurses and midwives, it is impossible to enhance intravenous fluid (IVF) administration practices and improve patient safety only by the sole efforts of nurses and midwives. As our search, studies to investigate intravenous fluid administration practices and its associated factors among nurses and midwives could not be found and there is little evidence on this topic in the country. Even though IVF administration practice is critical procedure in hospital, it is masked under the general medication administration practices. So, most of the time nurses and midwives do not give serious attention. Therefore, this study was aimed to assess IVF administration practices and associated factors among nurses and midwives working in public hospitals of West Shewa Zone, Oromia Region, Ethiopia.

## 2 Methods

An Institution-based cross-sectional study was conducted among nurses and midwives working in public hospitals of West Shewa zone from March 01 to 31, 2019. In this zone, there are 520 health posts, 92 health centers, and 8 hospitals. These mentioned above health institutions serves an estimated at 2,058,676 a total population of which 1,028,501 are males [18]. According to the report from human resource of the respected hospitals, the total number of nurses and midwives were 490.

### 2.1 Study population

All nurses and midwives working in West Shewa Zone public hospitals were the source population and all nurses and midwives working in units where intravenous fluid administrations have given in West Shewa Zone public hospitals during the study period were the study population. Nurses working in the Medical Ward, Surgical Ward, Pediatrics ward, AICU, NICU, Emergency Ward, Delivery ward and Gynecology, and Obstetrics wards were included and Nurses and Midwives who were not available during the data collection period were excluded.

### 2.2 Sample size determination

The sample size for the study was determined by using the single population proportion formula with a 95% confidence interval, a margin of error 5%, and prevalence of nurse's practice of accurately adjusting the flow rate is 43.33% [11].

By using the formula: n = (Z α/2)2 p (1-p) = (1.96) 2 × 0.433(1–0.433) = 377

1 $$D^{2}\;\left(0.05\right)$$

The calculated sample size was 377. By adding 10% non-response rate the total sample size was 415. Where: N = the required minimum sample size, P = the prevalence of IVF administration practices (in this case practice of accurately adjusting the flow rate), D = margin of error, Z α/2 = critical value at 95% confidence level (1.96).

## 3 Sampling procedures

First, all eight hospitals found in West Shoa Zone were included in the study to get the desired number of nurses. The required sample size was proportionally allocated for those hospitals then selected using simple random sampling. However, the IV fluid administration practices was recorded through a self-reported survey for all of the participants and was also observed for 10% of the participants (415) 42 nurses and midwives were observed on IV fluid administration practices using a checklist. They were selected from AURH and AGH purposely. A number of nurses and midwives in the two hospitals were 210. Then 42 nurses and midwives were proportionally allocated for the two hospitals (AURH = 24 and AGH = 18). Finally, those study participants were selected by simple random sampling.

## 4 Data collection methods

### 4.1 Data collection tool

Semi-structured self-administered questionnaire and observational checklist prepared in English were used as a tool to collect data. The questionnaire was translated from the English language to the local language (Afaan Oromoo and Amharic) translated back to English by two different language experts. The questionnaire consists of 4 sections was adapted with modification from similar previous related studies and IV therapy guidelines [10,11,16,19,20].

The first section was dealing with socio-demographic characteristics of respondents including sex, age, and education qualification. The second section consists of questions related to other factors associated with the practices of IV fluid administration including the length of services, attendance of in-service training, working unit, work overload, time shortage, supervision, Incentives and promotion and Fair distribution of nurses at wards. The third section consists of 10 questions designed to assess knowledge of nurse related to IV fluid administration practices. The fourth section consists of questions related to practices of IV fluid administration. Observation checklist consists of 10 items to assess the practice of nurses regarding IV fluid administration were also used. In the knowledge questionnaire, each correct answer scores one and each wrong answer scores zero. The data collectors observed each respondent for each performed action and the participants were scored 1 (met) when the action was done or not applied were scored 0 (unmet).

Data were collected by 6 diploma nurses and 2 BSc nurse supervisors who were working in other institutions and fluent speakers of both Amharic and Afaan Oromo languages. Having ethical clearance from the IHRERC and the permission from the study settings to conduct the study, the researcher first introduced and explained the need and the purpose of the study to hospital managers. Afterward, the data collectors met staff nurses to explain the objectives of the study and procedures of data collection. Furthermore, respondents were informed that the study is not an audit of their work and would not be linked with their performance appraisal. Next, the participants who agreed to participate in the study were requested to sign a consent form. Data were collected only from those participants signed the consent using pretested self-administered questionnaire and observation checklist from March 1 to 31, 2019.

To minimize bias observation took place first for those respondents selected in both assessments (Self-administered questionnaire and observation checklist) when they were practicing. The data collectors explained to participants that they were observed while they are performing IVF administration. However, the exact time and detail of the checklist were not explained for respondents. The researcher was available when required during the filling of the questionnaire physically or through the phone to explain and answer questions raised from the respondents. The data collectors, supervisors, and researcher made a discussion on the data collection process. The questionnaire was checked by the supervisors on a daily basis for completeness.

### 4.2 Study variables

#### 4.2.1 Dependent (outcome) variable

⁃IV fluid administration practices

### 4.3 Independent (explanatory) variables

⁃ Socio-demographic characteristics: sex, age, education qualification.
⁃ Work-related factors: work unit (ward), time shortage, work overload, length of services.
⁃ Organizational factors: supervision, training, incentives and promotion, distribution of available nurses in the ward.
⁃ Knowledge of nurses.

### 4.4 Data quality control

Two days training was given for data collectors and supervisors on the data collection process. Data collectors were supervised by the supervisors and reported to the principal investigator on a daily basis. Prior to the actual data collection, the questionnaires were pre-tested among 5% of the sample size in Sibu Sire hospital, a nearby zone to the study area. Tool reliability and validity were estimated. The result of reliability tests showed that Cronbach's alpha for question was 0.79 on pre-test. After pre-test and revision from experts, some modifications were incorporated for its validity. Then necessary comments and feed backs were incorporated into the final tools. To keep completeness and consistency, data collectors were closely supervised during the data collection process by the supervisor. The principal investigator (PI) supervised the correct implementation of the procedure.

## 5 Methods of data processing and analysis

All self-administered questionnaires were checked manually for their completeness and consistency. Double data entries were done by two individuals using the Epi data vs3.1 software to minimize errors. Data were exported to the SPSS statistical package vs23 then checked, cleaned and analyzed. Descriptive statistics were done to describe the relevant variables and presented in tables. Bivariable analysis was carried out to identify variables that were significantly associated with IVF administration practices among nurses and midwives. Those variables in the bi-variable analysis whose p-value is less than 0.25 were included in multiple logistic regression analysis. No multi co-linearity of independent variables was detected as standard error < 1. Then multiple logistic regression analysis was run for those factors that showed a statistically significant association in the bivariable analysis and investigated independent predictors by controlling for possible confounders. Hosmer- Lemeshow = 0.42 which is insignificant and Omnibus = 0.00 which is found to be significant. Finally, variables whose p-value less than 0.05 in the logistic regression were considered as statistically significantly associated. For measuring the strength of the association between the outcome and independent variables, Crude Odds Ratio (COR) and Adjusted Odds Ratio (AOR) along with 95% Confidence interval (CI) were calculated. For the observation part, descriptive statistics were calculated. Then percentage and mean were presented in tables and graphs.

### 5.1 Ethical consideration

The study was conducted following approval by the Haramaya University, College of Health and Medical Sciences, Institutional Health Research Ethics Review Committee (IHRERC). The official letter was received from Haramaya University to the respected hospitals. An informed voluntary written and signed consent was obtained from directors of the hospitals and all study subjects for their participation after the nature of the study was fully explained to them. Only those who signed written consent participated in the study and confidentiality of responses was maintained throughout the research process by giving the code for participant-filled questionnaires. The entire study participants were informed that data was kept private and confidential and used only for research purpose. The participants were also assured that they have the right to refuse or withdraw if they were not comfortable at any time. Personal privacy and cultural norms were respected. Awareness of IVF administration practices and its consequences was provided to all nurses and midwives after data collection was completed. Personal identifiers were not included in the written questionnaires to ensure participants’ confidentiality.

## 6 Results

### 6.1 Socio-demographic characteristics and work-related factors of the study participants

A total of 396 study participants were participated in the study with 95.42% response rate. The majority of them, 237 (59.8%) were males. In this study participants age were ranged between 23 and 55 years with a mean age of 28.81 (SD = ± 3.95) years. The majority, 351 (88.6%) of respondents had bachelor degrees and above. Three hundred fifty-eight (90.4%) were working in non ICU wards while 38 (9.6%) were working in ICU (adult and neonatal) wards. The highest percentage, 280(70.7%) of them had a length of service five and below years followed by 62(15.7%) who worked 6–10 years. Majority of respondents 254(64.1%) reported that there is a time shortage to perform complete IVF administration practices. In the same way, 239(60.4%) of them responded that there is work overload (Table 1).

**Table 1:** Socio-demographic characteristics among nurses and midwives working in public hospitals of West Shewa zone, Oromia Region, Ethiopia, 2019 (n = 396).

Variables	Characteristics	Frequency	Percent
Sex	Male	237	59.8
	Female	159	40.2
Age	23–29	274	69.2
	30–39	113	28.5
	>/40	9	2.3
Level of education	Diploma	45	11.4
	BSc. and above	351	88.6
Working experience in year	≤5	280	70.7
	6–10	62	15.7
	≥11	54	13.6
Working unit	ICU	38	9.6
	Non-ICU	358	90.4
Time shortage	Yes	254	64.1
	No	142	35.9
Work overload	Yes	239	60.4
	No	157	39.6

### 6.2 Organizational characteristics of the study participants

The majority, 298 (75.3%) of respondents claimed that they had not received training while 98(24.7%) of them had received on jobs or off jobs training about IV fluid administration in any form. Regarding their distribution in wards, 360 (90.9%) of respondents reported not fair. More than half of the respondents 244(61.6%) indicated that there was supervision by senior nurses and nurse heads. The majority, 308(77.8%) of respondents responded that there were incentives and promotion in different ways (Table 2).

**Table 2:** Organizational characteristics among nurses and midwives working in public hospitals of West Shewa zone, Oromia Region, Ethiopia, 2019 (n = 396).

Variables	Characteristics	Frequency	Percent
**Training**	Yes	98	24.7
	No	298	75.3
**Supervision**	Available	244	61.6
	Not-available	152	38.4
**Incentives and promotion**	Yes	308	77.8
	No	88	22.2
**Nurse distribution**	Fair	36	9.1
	Not fair	360	90.9

### 6.3 Knowledge of nurses and midwives regarding IV fluid administration practices

**Fig. 1:**
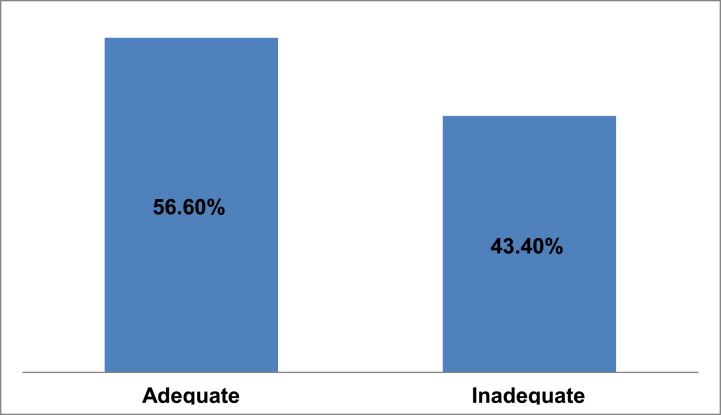
Knowledge about IV fluid administration practices among study participants of West Shoa zone, Ethiopia, 2019.

This study demonstrates responses of the participant's on knowledge affect IVF administration practices using 10 questions. The mean (±SD) knowledge score of respondents on IVF administration practices was 5 (±3.75). Among 396 respondents majority 224 of them had adequate knowledge 56.6% (95% CI: 51.8%–61.4%) about IVF administration while 172 had inadequate knowledge 43.4% (95% CI: 38.6%–48.2%) (Fig. 1).

### 6.4 Self-reported practices of IV fluid administration among nurses and midwives

This study shows responses of the participant's on IVF administration practices using 10 questions. The mean (+SD) practice score of respondents on IVF administration practices was 38.9 (±5.24). Adjusting flow rate of fluid given, 228(57.6%), checking the amount of fluid, 180(45.5%) and type of fluid, 137 (34.6%) were tasks always practiced by respondents. Of the study participants, 115(29%) checked vital sign and 128(32.3%) documented time fluid administration started sometimes. On the other hand, 43(10.9%) of respondents never flushed IV tube with heparinized normal saline after they had administered the fluid (Table 3).

**Table 3:** Self-reported practices of IV fluid administration among respondents in public hospitals of West Shewa zone, Oromia Region, Ethiopia, 2019 (n = 396).

Items	Level of practice by frequency and percentage				
	Not at all	Very seldom	Sometimes	Nearly always	Always
Check the amount of fluid against doctor's	9	4	64	139	180
orders	(2.3)	(1.0)	(16.2)	(35.1)	(45.5)
Check the type of fluid against doctor's	4	6	107	142	137
orders	(1.0)	(1.5)	(27)	(35.9)	(34.6)
Check V/S before IVF administration	3	7	115	169	102
	(8)	(1.8)	(29)	(42.7)	(25.8)
Document the prescribed fluid on chart	3	16	96	187	94
	(8)	(4)	(24.2)	(47.2)	(23.7)
Document the time fluid administration	6	13	128	156	93
started	(1.5)	(3.3)	(32.3)	(39.4)	(23.5)
Label the date the fluid is opened/given	8	23	124	166	75
	(2.0)	(5.8)	(31.3)	(41.9)	(18.9)
Adjust the flow rate accurately as prescribed	5	1	40	122	228
	(1.3)	(0.3)	(10.1)	(30.8)	(57.6)
Flush the tubing with heparinized normal	43	34	138	129	52
saline at the end of the administration of	(10.9)	(8.6)	(34.8)	(32.6)	(13.1)
drugs, blood, and electrolytes					
Document the amount of fluid infused	6	18	109	183	80
	(1.5)	(4.5)	(27.5)	(46.2)	(20.2)
Document the additives which are added to	9	21	84	189	93
the fluid	(2.3)	(5.3)	(21.2)	(47.7)	(23.5)

### 6.5 Overall self-reported IVF administration practices

**Fig. 2:**
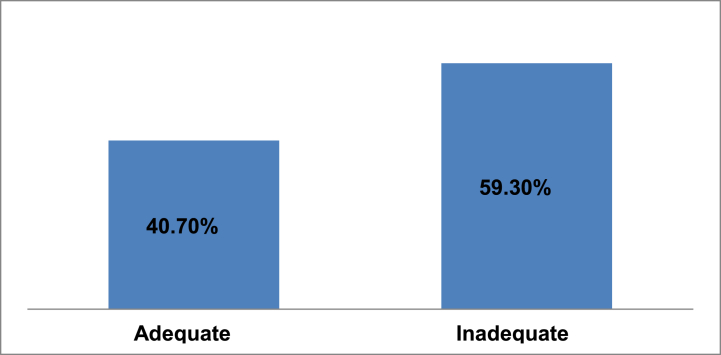
The overall self-reported IV fluid administration practices among nurses and midwives working in public hospitals of West Shoa Zone, Ethiopia.

The overall inadequate IVF administration practices among nurses and midwives were 235(59.3%) [95% CI = (54.7%–64.5%)] while the adequate practices were 161(40.7%) [95% CI = (35.5–45.3)] (Fig. 2).

### 6.6 Factors associated with self-reported IVF administration practices of nurses and midwives

In bivariable model, practices of respondents on IVF administration were significantly associated with knowledge, in-service training, incentives and promotion, supervision, education qualification, length of service, time shortage, nurse distribution in wards and workload. To the contrary, there was no significant association of age, sex and work unit with IVF administration practices among nurses' and midwives’ from the model.

In multivariable logistic regression analysis, among variables those significantly associated with outcome variable in bivariable analysis, knowledge, training, incentives and promotion and availability of supervision were retained as determinant factors for IVF administration practices. Time shortage and education qualification were significantly but negatively associated with IVF administration practices. On the other hand work overload, nurse distribution in wards and length of services were factors not significantly associated with IVF administration practices. The odds of having inadequate practices were 1.7 times more likely among respondents who had not been received training as compared to those who did receive[AOR 1.73; 95% CI=(1.04–2.86)]. Participants who reported incentives and promotion is not available were 2.1 times more likely to have inadequate IVF administration practices as compared to those who responded yes [AOR 2.12; 95% CI = (1.19–3.62)]. Respondents who responded there is no supervision were 1.8 times more likely to have inadequate IVF administration practices than those who responded there is supervision [AOR 1.84; 95% CI = (1.14–2.99)]. The odds of having inadequate practices were 2.1times more likely among respondents with inadequate knowledge as compared to those with adequate knowledge [AOR 2.13; 95% CI = (1.36–3.36)] (Table 4).

**Table 4:** Factors associated with self-reported IV fluid administration practices among respondents working in public hospitals of West Shewa Zone, Oromia, Ethiopia, 2019.

Characteristics		Practice level		COR(95%CI)	AOR(95%CI)
		Inadequate	Adequate		
Education qualification	Diploma	17	28	0.37(0.20–0.70)	0.385(0.20–0.79)
	BSc and above	218	133	1.00	1.00
Length of Service	≤5	182	98	1.724(0.96–3.10)	
	6–10	25	37	0.627(0.30–1.31)	
	≥11	28	26	1.00	1.00
Training	No	183	108	1.727(1.10–2.71)	**1.726(1.04**–2.86)*
	Yes	52	53	1.00	1.00
Time shortage	Yes	135	119	0.476(0.31–0.74)	0.59(0.36–0.96)
	No	100	42	1.00	1.00
Nurse distribution	Not fair	205	155	0.265(0.11–0.65)	
	Fair	30	6	1.00	1.00
Incentives and promotion	No	62	26	1.861(1.12–3.10)	**2.08(1.19**–3.62)**
	Yes	173	135	1.00	1.00
Supervision	Not available	101	51	1.626(1.067–2.476)	**1.841(1.14**–2.99)*
	Available	134	110	1.00	1.00
Work overload	No	149	90	1.367(0.91–2.06)	
	Yes	86	71	1.00	1.00
Knowledge	Inadequate	121	51	2.289(1.51–3.48)	**2.13(1.36**–3.36)**
	Adequate	114	110	1.00	1.00

**Notes:** * = p-value ≤ 0.05, ** = p-value ≤ 0.01, CI = confidence intervals, COR = crude odds ratio, AOR = Adjusted Odds Ratio, 1 = reference.

### 6.7 Analysis of observational checklist assessment

Observational checklist assessment on IVF administration practices was done among 42 study participants of two hospitals using 10 items. The mean (±SD) practice score of participants on IVF administration practices was 17.05 ± 1.99. Almost all participants did not meet the criteria for each IVF administration practice. Accordingly, only 38.1%, 33.3% and 31% of participants check the type of fluid, adjusted flow rate of fluid administered and flush tube with heparinized normal saline during observation respectively. On the other hand, the majority (73.8%) of the participants did not check the amount of fluid against doctor's orders. In the same way, 73.8% of participants did not document the amount of fluid infused and the time fluid administration was started (Table 5).

**Table 5:** Observational checklist assessment on Practices of IV fluid administration among selected participants in public hospitals of West Shoa Zone, Oromia, Ethiopia, 2019(n = 42).

Characteristics	Response	Frequency	Percentage
Check the amount of fluid against Doctors' order	Met	11	26.2
	Unmet	31	73.8
Check the type of fluid against Doctors' order	Met	16	38.1
	Unmet	26	61.9
Accurately adjust the flow rate	Met	14	33.3
	Unmet	28	66.7
Check V/S before IVF administration	Met	12	28.6
	Unmet	30	71.4
Document the prescribed fluid on chart	Met	12	28.6
	Unmet	30	71.4
Document the time started	Met	11	26.2
	Unmet	31	73.8
At the end of the administration of drugs and electrolytes flush the tubing with heparinized normal saline	Met	13	31
	Unmet	29	69
Mention the amount of fluid infused	Met	11	26.2
	Unmet	31	73.8
Document the additives which are added to the fluid	Met	13	31
	Unmet	29	69
Label the date in which the fluid bottle is opened	Met	11	26.2
	Unmet	31	73.8
**Overall practice level**	**Adequate**	**10**	**23.8**
	**Inadequate**	**32**	**76.2**

### 6.8 An overall score of the observational checklist assessment

The overall observational checklist assessment scores ranged from 0 to 10. The score 80% and above was considered as adequate IVF administration practices. Thus, 76.2% of the participants had a score below cut point (80%) and considered as inadequate IVF administration practices. This poor performance is particularly due to the fact that participants did not document what they did for the patients.

## 7 Discussion

In this study, more than half of the respondents involved in self-reported assessment had inadequate IVF administration practices, and out of 42 participants observed using observational checklist assessment, about three-fourth of them had inadequate IVF administration practices. In both cases, the majority of respondents’ IVF administration practices were inadequate. Though they are supporting each other, the result obtained by the observational checklist assessment was higher than the result obtained by self-reported assessment. The difference between the two results can be due to nurses and midwives may report what they have not done and this bias might be decreased by observation. Observation was also done only once for one respondent. This could result in higher inadequate IVF administration practices in the case of observation. Inadequate knowledge of respondents, being un-trained, absence of supervision and incentives and promotion for nurses and midwives were factors significantly and positively associated with inadequate IVF administration practices.

This study found that the overall inadequate IVF administration practices obtained by self-reported and observational checklist assessment were about 60% and 76% respectively which were high. These results are higher than the descriptive observational study done in Zambia [11] and Pakistan, Lahore University Hospital [10] which revealed that majority of nurses did not accomplish the procedure as a result of this IVF administration practice was inadequate. The possible explanation for these differences could be due to the differences in the study design which is pure observation in the previous studies while cross-sectional study design was used in this case; sampling technique in which census was used in the previous studies. It is also due to sample size used which is small in previous studies and study setup where the previous studies were conducted in relatively advanced hospitals. Knowledge difference among nurses in previous studies and this study could be another reason for the discrepancy. The previous studies were specific to ICU whereas this study whereas this study is based on both ICU and non- ICU. Moreover, it could be due to poor attention given to IV fluid administration practices in this case. This leads to poor nursing care that may cause complications and mortality as the wrong amount and types of fluids can be given to patients.

This study finding revealed that study participants who reported no incentives and promotion were almost two times more likely to have inadequate practice than those who responded yes. This is supported by a study done in Southern Ghana which revealed that poor motivation in the form of promotion and incentives has a significant effect on the practice of nursing care documentation. This could be due to the reason that as nurses and midwives had incentives and promotion their performance got improved. Consequently, the quality of nursing care increases so does IVF administration practices. In general, patient suffering due to inappropriate fluid composition decreases.

Inadequate IVF administration practices were more likely among nurses and midwives who had not received training as compared to those who received training. This is supported by the study done in Pakistan [10] and Aksehir, Konya [21] which dictated inadequate training is a significant factor that impairs the fluid balance documentation. The result of this study is also supported by the study done at England; Milton Keynes University Hospital that identified one of the major reasons for incomplete fluid balance charts was lack of training [22]. This may be due to the reason that regular in-service training enables nurses and midwives to increase their knowledge and implementation skills. This increases correct and appropriate IVF administration practices which prevent complications that may arise from fluid imbalance. This, in turn, increases patient safety.

According to this study nurses and midwives who had inadequate knowledge on IVF administration practices were more likely to have inadequate practices as compared to those with adequate knowledge about it. This is supported by the study done in Zambia that showed a positive relationship between knowledge and practice of the nursing staff regarding 10.13039/100012825IVF administration practices [20]. The possible reason may be due to the fact that adequate knowledge improves the confidence and readiness of nurses and midwives to perform their routine activities correctly. Knowledge also helps nurses and midwives to understand the consequences of incorrect and inappropriate IVF administration on patients. Thus, they administer IVF correctly to patients. Ultimately decreases morbidity and health care costs for patients.

This study found that nurses and midwives who had not been supervised on IVF administration were more likely to have inadequate practices than those who had been supervised. This is supported by the study done in 10.13039/501100005068Jimma University Specialized Hospital, Ethiopia [23] which showed that supervision by more experienced nurses or other relevant professionals in regular intervals was found to be helpful in minimizing medication administration errors. This study is also supported by a study done in Baghdad [12] which noticed that deficient monitoring and supervision of patients on 10.13039/100012825IVF by specialists leads to a quite high ratio of using wrong 10.13039/100012825IVF. This may be due to the reason that supervision and support by experienced professionals’ increases the concern of nurses and midwives to perform correct 10.13039/100012825IVF administration. This results in good nursing care that decreases the morbidity and mortality of patients from inappropriate IVF administration.

Time shortage and being diploma holders were significantly but negatively associated with inadequate IVF administration practices. On the other hand work overload, nurse distribution in wards and length of services were factors not significantly associated with IV fluid administration practices.

The strengths of the study were eight government hospitals were included in the study to make the study representative. This study used primary data. The observational checklist was used to support data collected by self-administered questionnaire. The major limitations of this study were collected with self-administered questionnaire; the study may be subjected to recall bias from the respondents. Only two hospitals (AURH and AGH) were included for observation was a limitation to the study.

## 8 Conclusion

Based on the finding of this study, the following are concluded: the result of this study indicated that overall IVF administration practices of nurses and midwives were inadequate. Inadequate knowledge, training, and absence of supervision by senior nurses and head nurse, and absence of incentives and promotion for nurses and midwives were the main factors affecting IVF administration practices.

## 9 Implication for nursing

Hospitalized patients are often administered with IVF. Nurses and midwives play an important role in IVF administration practices. Based on the findings of this study nurses and midwives had been practicing IVF administration inadequately. But nurses and midwives should perform IVF administration practices correctly. When nurses and midwives follow correct procedures of IVF administration they could evaluate their practice. This would help patients to have a good outcome. It could also help nurses and midwives to identify patients at risk of developing fluid complications so that can develop an individualized treatment plan. This would include checking amount and types of fluid ordered, adjusting the drop rate and finally documenting what has been done for patients. Understanding these tasks will facilitate appropriate and correct IV fluid administration and prevent complications that may happen to patients. This study could also identify factors significantly affecting IV fluid administration practices. Therefore, as stakeholders act on those identified factors it would enhance nursing care so does IV fluid administration practices.

## 10 Recommendations

Based on the study findings, the following recommendations were forwarded for responsible bodies; Health service managers need to identify factors associated with IV fluid administration practices and take action on it. Thus, Stakeholders of nursing services such West Shoa Zone health office and the director of nursing services in each hospital had better provide effective refresher courses on IV fluid administration practices by expert nurses at regular intervals and the results should be evaluated.

Hospital administrators should also ensure sufficient and regular supervision and support in each unit. Incentives and promotion for nurses and midwives should be available.

For nurses and midwives: Nurses and midwives need to acquire the necessary knowledge on IV fluid administration in order to further improve nursing practice in this area. Nurses, who have better knowledge, should also teach their respective colleagues who had deficits for the betterment of nursing care.

For Researchers:

Other researchers need to do further study on this topic using qualitative study design.

## Consent for publication

Not applicable.

## Author contribution statement

Milion Teshome and Meseret Robi Tura: Conceived and designed the experiments; Analyzed and interpreted the data. Biftu Geda, Tesfaye Assebe and Lema Mideksa: Analyzed and interpreted the data. Milion Teshome, Biftu Geda, Tesfaye Assebe, Lema Mideksa and Meseret Robi Tura: Performed the experiments; Contributed reagents, materials, analysis tools or data; Wrote the paper.

## Data availability statement

Data included in article/supplementary material/referenced in article.

## Declaration of interest's statement

The authors declare no conflict of interest.

## Declaration of competing interest

The authors declare that they have no known competing financial interests or personal relationships that could have appeared to influence the work reported in this paper.

## Appendix A Supplementary data

Operational Definitions.docx Operational Definitions.docx

Questionnaire.docx Questionnaire.docx
