# Corrigendum to “Intravenous fluid administration practice among nurses and midwives working in public hospitals of central Ethiopia: A cross-sectional study” [Heliyon Volume 9, Issue 8, August 2023, Article e18720]

**DOI:** 10.1016/j.heliyon.2025.e43357

**Published:** 2025-04-29

**Authors:** Million Teshome, Biftu Geda, Tesfaye Assebe Yadeta, Lema Mideksa, Meseret Robi Tura

**Affiliations:** a Department of Nursing, College of Medicine and Health Sciences, Ambo University, Ethiopia; b School of Public Health, College of Health Sciences and Medicine, Haramaya University, Ethiopia; c School of Nursing and Midwifery, College of Health Sciences and Medicine, Haramaya University, Ethiopia

In this article, the authors omitted the approval number and date that approval was obtained from their ethical approval statement, which is required by the policies of the journal. The authors confirm that ethical approval was obtained from the Haramaya University, College of Health and Medical Sciences, Institutional Health Research Ethics Review Committee (IHRERC) for the research under the following reference number: IHRERC/013/2019 dated February 19th^,^ 2019.

The ethical statement in section *5.1. Ethical consideration* of the publication is below:

*The study was conducted following approval by the Haramaya University, College of Health and Medical Sciences, Institutional Health Research Ethics Review Committee (IHRERC).*

The ethical statement has been updated to include the ethical approval number and date that approval was obtained.

The correct version of the ethical statement should be as below:

*The study was conducted following approval by the Haramaya University, College of Health and Medical Sciences, Institutional Health Research Ethics Review Committee (IHRERC), with the approval number* IHRERC/013/2019 dated February 19th^,^ 2019.

The ***authors*** apologize for the errors.

## Declaration of competing interest

The authors declare no conflict of interest.

