# Supplementary material for: Intravenous fluid administration practice among nurses and midwives working in public hospitals of central Ethiopia: A cross-sectional study
Source: Heliyon. 2023 Jul 31;9(8):e18720. doi: 10.1016/j.heliyon.2023.e18720 (PMC10412755; doi:10.1016/j.heliyon.2023.e18720)
Supplement: Operational Definitions.docx [file mmc1.docx]

**Operational Definitions**

| **Intravenous fluid administration practice** | It was measured through practice related questionnaires and an observational schedule. The practice level of nurses and midwives was measured by the total number of response to 10 items related to IV fluid administration with a minimum score of 0 and a maximum of 50. For analysis of the outcome variable, the value 80% and above was coded as” 0” and below 80% was coded as ‟ 1”. “Inadequate” Practice level is when the respondents scored below 80% for practice related questions otherwise adequate. |
| --- | --- |
| **Knowledge** | Knowledge of nurses and midwives was measured by the total number of correct answers to 10 items on knowledge related to practice with a minimum score of 0 and a maximum of 10. The independent variable, knowledge level of the respondent, value 75% and above was coded as‟ 1‟ and value below 75% was coded as ‟0‟. “Adequate” knowledge is when nurses and midwives scored 75% and above on knowledge items otherwise inadequate. |
| **Work overload** | Refers to the situation in which one ICU trained nurse provides nursing care services for more than 2 beds in ICU and when one nurse provides nursing care for more than 6 patients in an inpatient service other than ICU and emergency per shift. |
| **Clinical supervision** | Refers to an ongoing formal process of professional support and learning with an experienced practitioner which enables individual practitioners to develop knowledge and competence in a clinical situation. |
| **Fair distribution of available nurses** | The distribution of nurses in different wards taking their skill mix and qualification in to consideration. |
