# Supplementary material for: Intravenous fluid administration practice among nurses and midwives working in public hospitals of central Ethiopia: A cross-sectional study
Source: Heliyon. 2023 Jul 31;9(8):e18720. doi: 10.1016/j.heliyon.2023.e18720 (PMC10412755; doi:10.1016/j.heliyon.2023.e18720)
Supplement: Questionnaire.docx [file mmc2.docx]

Questionnaire:

This questionnaire comprises of four sections: each section has a stem of questions related to IV fluid administration practices. The questions aim to establish the factors that influence your IV fluid administration practices in your hospitals.

**Instructions:** Please do not write your name on the questionnaire. Kindly attempt all the questions.

Date

Name of data collector Name of supervisor Questionnaire Code number

Signature _ Signature

Please! Read each statement carefully and **encircle** to the most appropriate answer. Total of eleven questions, each question carries one mark.

**Socio-demographic data of the respondent.**

| S.N | List of questions | Options | Codes | Skip to Q |
| --- | --- | --- | --- | --- |
| Q001 | Sex | Male | 1 |  |
|  |  | Female | 2 |  |
| Q002 | Age in complete years | **---------------**years |  |  |
| Q003 | Educational qualification | Diploma/Level IV | 1 |  |
|  |  | BSc in nursing | 2 |  |
|  |  | MSc in nursing | 3 |  |

**Organizational and work-related questions.**

| S. N | List of questions | Options | Codes | Skip to Q |
| --- | --- | --- | --- | --- |
| Q004 | Work experience in nursing | **---------------**year(s) |  |  |
| Q005 | Work unit/ ward | Medical ward | 1 |  |
|  |  | Surgical ward | 2 |  |
|  |  | Gyn/Obs ward | 3 |  |
|  |  | Emergency unit | 4 |  |
|  |  | NICU | 5 |  |
|  |  | AICU | 6 |  |
|  |  | Pediatric ward | 7 |  |
|  |  | Delivery ward | 8 |  |
| Q006 | Have you attended any in-service | Yes | 1 |  |
|  | training on IVF/medication | No | 2 |  |
|  | administration? |  |  |  |
| Q007 | How often does supervision take place | No supervision | 1 |  |
|  | at your ward? | Once per month | 2 |  |
|  |  | Once per two month | 3 |  |
|  |  | Once per six month | 4 |  |
| Q008 | On average for how many patients do  you give care per day? | **-----------**patients |  |  |
| Q009 | Is there a shortage of time on | Yes | 1 |  |
|  | performing IVF administration | No | 2 |  |
|  | correctly? |  |  |  |
| Q010 | Is the distribution of available nurses | Fair | 1 |  |
|  | on ward fair or not? | Not fair | 2 |  |
| Q011 | Does your organization give any form | Yes | 1 |  |
|  | of incentives and promotion? | No | 2 |  |

**A structured questionnaire on knowledge**.

Please read each statement carefully and **encircle** the appropriate answer. Total often questions (**N.B**. **More than one answer is possible)**.

| S.N | List of | questions | | Options | Codes | Skip  to Q |
| --- | --- | --- | --- | --- | --- | --- |
| Q301 | Which of the following is important in IVF administration? | | | Adjusting the flow rate Checking the types of fluid  Labeling the date fluid is given | 1  2  3 |  |
| Q302 | What is the most used physiological | | | Normalsaline | 1 |  |
|  | intravenous fluid? | | | isolyte P | 2 |  |
|  |  | | | ringerlactate | 3 |  |
|  |  | | | 3% saline | 4 |  |
| Q303 | Which | of the following | fluid is | Albumin | 1 |  |
|  | crystalloid? | | | Ringer lactate | 2 |  |
|  |  | | | Plasma | 3 |  |
| Q304 | Which one of the following is an | | | Fluid replacement | 1 |  |
|  | indication of IV fluid administration? | | | As maintenance | 2 |  |
|  |  | | | Resuscitation | 3 |  |
| Q305 | Which of the following vital signs | | | Temperature | 1 |  |
|  | must be checked prior to IVF administration? | | | Pulse rate Respiration | 2  3 |  |
| Q306 | IVF | prescription should | include | Type of fluid to be |  |  |
|  |  | | | administered | 1 |  |
|  |  | | | The rate of fluid to be |  |  |
|  |  | | | administered | 2 |  |
|  |  | | | The volume of fluid to |  |  |
|  |  | | | be administered | 3 |  |

| Q307 | Which one of the following should be included in the IVF administration documentation? | Time fluid is given Amount of fluid Type of therapy | 1  2  3 |  |
| --- | --- | --- | --- | --- |
| Q308 | When the Doctor orders to administer 1000ml of NS solution over 4hours, how many drops of fluid is given per minute? | 73.33  83.33  333.33  250 | 1  2  3  4 |  |
| Q309 | Documentation should be | Legible Timely Complete | 1  2  3 |  |
| Q310 | What is the purpose of flushing IV tube with heparinized normal saline? | To prevent clotting of blood in the IV tube To prevent bleeding | 1  2 |  |

Section IV. IV fluid administration practices related questionnaire.

| **S. No** | **Questions** | Not at all  (1) | Very seldom  (2) | Some- times  (3) | Mostly  (4) | Always  (5) |
| --- | --- | --- | --- | --- | --- | --- |
| Q401 | I check the amount of fluid against  doctor’s orders |  |  |  |  |  |
| Q402 | I check the type of fluid against doctor’s  orders |  |  |  |  |  |
| Q403 | I check V/S before IVF administration |  |  |  |  |  |
| Q404 | I document the prescribed fluid on chart |  |  |  |  |  |
| Q405 | I document the time fluid administration  started |  |  |  |  |  |
| Q406 | I label the date the fluid is opened/ given |  |  |  |  |  |
| Q407 | I adjust the flow rate accurately as  prescribed |  |  |  |  |  |
| Q408 | I flush the tubing with heparinized normal saline at the end of the  administration of fluids. |  |  |  |  |  |
| Q409 | I document the amount of fluid infused |  |  |  |  |  |
| Q410 | I document the additives which are  added to the fluid |  |  |  |  |  |

THANK YOU FOR YOUR COOPERATION!

**OBSERVATIONAL SCHEDULE**

Evaluation of IV fluid administration practices. Instructions for the data collectors:

No nurse name should appear on the checklist.

Put the letter “X” in the box next to the observed item (under **Met** when the procedure is performed or under **Unmet** when the procedure is not performed).

All information collected should be kept confidential.

Hospital name

| **S. No** | **Check list** | **Met** | **Not met** |
| --- | --- | --- | --- |
| 01 | Check the amount of fluid against doctors orders |  |  |
| 02 | Check the type of fluid against doctors orders |  |  |
| 03 | Accurately adjust the flow rate |  |  |
| 04 | Check V/S before IVF administration |  |  |
| 05 | Document the prescribed fluid on chart |  |  |
| 06 | Document the time started |  |  |
| 07 | At the end of the administration of drugs and electrolytes  flush the tubing with heparinized normal saline |  |  |
| 08 | Mention the amount of fluid infused |  |  |
| 09 | Document the additives which are added to the fluid |  |  |
| 10 | Label the date in which the fluid bottle is opened |  |  |
